# Disability-adjusted life years due to chronic and hidden hunger under food system evolution with climate change and adaptation to 2050

**DOI:** 10.1093/ajcn/nqab101

**Published:** 2021-05-20

**Authors:** Timothy B Sulser, Robert H Beach, Keith D Wiebe, Shahnila Dunston, Naomi K Fukagawa

**Affiliations:** International Food Policy Research Institute, Environment and Production Technology Division, Washington, DC, USA; RTI International, Environmental Engineering & Economics Division, Research Triangle Park, NC, USA; International Food Policy Research Institute, Environment and Production Technology Division, Washington, DC, USA; International Food Policy Research Institute, Environment and Production Technology Division, Washington, DC, USA; US Department of Agriculture, Agricultural Research Service, Beltsville Human Nutrition Research Center, Beltsville, MD, USA

**Keywords:** International Food Policy Research Institute's IMPACT model, disability-adjusted life years (DALYs), quantitative foresight modeling, food and nutrition security, food system, health outcomes, climate change, micronutrient deficiencies, undernutrition

## Abstract

**Background:**

Climate change presents an increasing challenge for food-nutrition security. Nutrition metrics calculated from quantitative food system projections can help focus policy actions.

**Objectives:**

To estimate future chronic and hidden hunger disability-adjusted life years (DALYs)—due to protein-energy undernutrition and micronutrient deficiencies, respectively—using food systems projections to evaluate the potential impact of climate change and agricultural sector investment for adaptation.

**Methods:**

We use a novel combination of a chronic and hidden hunger DALY estimation procedure and food system projections from quantitative foresight modeling to assess DALYs under alternative agricultural sector scenarios to midcentury.

**Results:**

Total chronic and hidden hunger DALYs are projected to increase globally out to 2050—by over 30 million compared with 2010—even without climate change. Climate change increases total DALY change between 2010 and 2050 by nearly 10% compared with no climate change. Agricultural sector investments show promise for offsetting these impacts. With investments, DALY incidence due to chronic and hidden hunger is projected to decrease globally in 2050 by 0.24 and 0.56 per 1000 capita, respectively. Total global DALYs will still rise because projected population growth will outpace the rate reduction, especially in Africa south of the Sahara. However, projections also show important regional reductions in total DALYs due to chronic (13.9 million in South Asia, 4.3 million in East Asia and the Pacific) and hidden hunger (7.5 million in East Asia and the Pacific) with investments.

**Conclusions:**

Food system projections to 2050 show a decreasing DALY incidence from both chronic and hidden hunger. Population growth is projected to outpace these improvements and lead to increasing total chronic and hidden hunger DALYs globally, concentrated in Africa south of the Sahara. Climate change increases per-capita chronic and hidden hunger DALY incidence compared with no climate change. Agricultural sector investments show the potential to offset the climate impact on DALYs.

## Introduction

Nearly 10% of the world's population is experiencing hunger or facing severe food insecurity (1), presenting significant challenges to achievement of the UN Sustainable Development Goal 2 of “Zero Hunger” by 2030. This is compounded by other obstacles that have arisen, from climate change (2) to tightened budgets for investments in food system improvements (3) to unforeseen shocks like the coronavirus disease 2019 (COVID-19) pandemic (4, 5). There is potential, however, to meet these challenges through policies that commit to increasing targeted investments for broad food system improvements (6–9). Modeling future food system scenarios (10) with connections across food production, food availability, and the health of people, the environment, and the economy (11, 12) is crucial to inform policymaking.

Using the International Food Policy Research Institute's International Model for Policy Analysis of Agricultural Commodities and Trade (IMPACT) (13), we projected disability-adjusted life years (DALYs) due to both chronic and hidden hunger following a published econometric analysis of historical and updated data from Gödecke et al. (14, 15). DALYs are defined as the sum of “years of life lost due to premature mortality and years lived with disability” (16). DALYs due to chronic and hidden hunger are defined, respectively, as those resulting from protein-energy undernutrition or those resulting from micronutrient deficiencies (iron, vitamin A, iodine, zinc, etc.) leading to stunting and wasting, anemia, immune system deficiencies, or impaired cognitive development. Both chronic and hidden hunger result primarily from shortages of major grains, which dominate diets around the globe. We use the IMPACT model linked to the Gödecke et al. (14, 15) econometric approach to project DALYs under alternative future climate and agricultural investment scenarios (17–19) to midcentury. These projections are useful for investigating the broader societal impacts of future shocks on human health that are mediated through the food system and the potential of policy and investment strategies to address them. This is analogous to the Lives Saved Tool (20) to assess specific health system interventions but keys off food system interventions that lead to quantifiable changes in food availability. Although the connections between climate change and hunger are deep and complex (21, 22), the objective is to introduce a generic approach that can be used to assess the human health outcomes of an array of food system shocks rather than all of the potential interactions between climate and hunger. This may be generalizable to other modeling efforts that generate estimates of future food availability, such as those in the Agricultural Model Intercomparison and Improvement Project (https://agmip.org/global-economics/) (10, 23).

Gödecke et al. (14) used historical data on a variety of determinants and indicators of the food system to estimate their impact on DALYs of chronic and hidden hunger [as accounted for in the Global Burden of Disease (GBD) database from the Institute for Health Metrics and Evaluation] (16). They specified a series of fixed-effects models looking at how food supply, food utilization, public health, and other factors affect the number of DALYs per capita attributed to chronic and hidden hunger.

Few other studies estimate food system impacts on future DALYs. Ishida et al. (24) estimated DALYs attributable to child undernourishment under climate change to midcentury, and Hasegawa et al. (25) extended this to 2100. Others have used DALYs to assess the impacts of a diet prescribed to achieve particular health or environmental goals (26, 27), of a targeted intervention for a narrow set of crops and nutrients such as biofortified maize in Zambia (28), or of a comparative static change from the potential impact of a posited future change imposed on today's diet (29).

## Methods

Using the Gödecke et al. (14) study, we assessed DALYs due to broader definitions of chronic and hidden hunger than in Ishida et al. (24) and Hasegawa et al. (25). We used the estimations based on historical data in the GBD database for analysis of future scenarios of the food system, projecting the potential health burden of chronic and hidden hunger in the future. The data flow used in this study is presented in **Supplemental Figure 1**. Focusing on Gödecke et al. (14) “Model 6” from their Table 3, DALYs from chronic hunger are due to inadequate food causing childhood undernourishment and inadequate supplies for the broader population measured by per-person kilocalorie availability of major food types (meat, fish, eggs, dairy, cereals, pulses, fruits and vegetables, and roots and tubers). DALYs from hidden hunger are due to deficiencies in micronutrients, including iodine, iron, zinc, and vitamin A, among others, associated with the same per-person kilocalorie availability of major food types as in the chronic hunger calculation. Per-capita gross domestic product (GDP) is a key proxy for a range of other socioeconomic factors and human development important for chronic and hidden hunger.

We specified a linear structural model form based on Gödecke et al. (14):

(1) $$\begin{eqnarray*}
{H_{xit}} = {\alpha _x} + {\beta _x}{\theta _{it}} + {\varepsilon _{xi}},
\end{eqnarray*}$$

where *H* is the natural logarithm of the relative burden of chronic or hidden hunger (*x*) in region *i* for time period *t*. The parameter α is the intercept term while β is a vector of coefficients specified at the global level for either chronic or hidden hunger. **Table 1** presents the α and β coefficients applied in our study. The β vector is applied to the region- and time-variant levels of per-capita GDP and food supplies (θ), summarized in **Table 2**. An iterative algorithm was used to determine a vector of regional-level composite error terms (ε) necessary to fit the regional estimates with the global specification. These regional ε terms are held constant through time and account for structural differences across regions that include factors such as health and sanitation, democratic governance and its stability, and education, among others. Constant regional ε terms allow focus on the core components of the structural model in θ and imply that regional factors not accounted for in our modeling—which might mean a region is either better or worse off than the simple global specification would indicate alone—remain unchanged in the future projections.

**TABLE 1:** Disability-adjusted life years (DALYs) estimation model specification based on detailed food supply (kcal/capita/d) disaggregation^1^

Characteristic	Chronic hunger	Hidden hunger
Log(GDP per capita, PPP)	−0.5548	−0.1672
Meat supply	−0.0010	−0.0008
Fish supply	0.0017	0.0015
Egg supply	−0.0077	−0.0037
Milk supply	−0.0012	−0.0005
Cereal supply	0.0002	−0.0001
Pulse supply	−0.0008	−0.0012
Fruit and vegetable supply	−0.0011	0.0004
Root and tuber supply	−0.0012	−0.0009
Constant	7.3453	4.5105

^1^Fixed effects; dependent variable logarithm of DALYs per 1000 capita. Chronic and hidden hunger DALYs are due to protein-energy undernutrition and micronutrient deficiencies, respectively. Reproduced from “Model 6” from Gödecke et al. (14); published open access under the CC BY-NC-ND license (http://creativecommons.org/licenses/BY-NC-ND/4.0/). GDP, gross domestic product; PPP, purchasing power parity.

**TABLE 2:** Summary of core projections from International Food Policy Research Institute's IMPACT model to drive calculations of disability-adjusted life years (DALYs)^1^

Characteristic	NoCC			CC		COMP	
	2010	2030	2050	2030	2050	2030	2050
Per-capita GDP (2005 US$ PPP)							
SSA	1974	3806	7789	3754	7499	3947	8028
SAS	2737	6981	13,881	6871	13,273	7321	14,476
EAP	8808	22,343	35,409	22,098	34,521	22,822	36,069
MENA	9956	17,089	26,045	17,001	25,656	17,424	26,568
LAC	9978	16,920	25,845	16,837	25,521	17,214	26,260
ECA	21,421	31,299	43,036	31,225	42,755	31,659	43,557
NAM	41,485	56,722	66,524	56,684	66,424	56,810	66,614
Meat supply (daily per-capita kilocalories)							
SSA	70	100	151	99	147	109	165
SAS	26	44	72	44	69	49	80
EAP	359	459	481	456	475	467	487
MENA	124	157	186	156	184	169	202
LAC	292	330	359	327	355	347	379
ECA	329	348	369	346	366	359	382
NAM	479	484	489	483	487	496	502
Fish supply (daily per-capita kilocalories)							
SSA	25	20	21	20	21	20	21
SAS	22	25	30	25	30	25	30
EAP	97	116	134	116	134	116	134
MENA	32	39	48	39	48	39	48
LAC	27	24	23	24	23	24	23
ECA	57	49	48	49	48	49	48
NAM	62	71	85	71	85	71	85
Egg supply (daily per-capita kilocalories)							
SSA	6	7	10	7	10	8	10
SAS	8	14	16	13	16	14	17
EAP	56	66	68	65	67	67	69
MENA	24	26	28	26	28	27	29
LAC	33	35	37	35	37	36	37
ECA	45	45	45	45	45	45	45
NAM	54	52	52	52	52	52	52
Milk supply (daily per-capita kilocalories)							
SSA	66	71	82	71	82	76	89
SAS	122	154	159	153	157	164	170
EAP	58	112	137	111	135	119	146
MENA	132	133	136	132	136	140	145
LAC	178	194	209	194	209	200	217
ECA	319	322	326	323	327	326	331
NAM	377	375	374	376	375	380	380
Cereal supply (daily per-capita kilocalories)							
SSA	1067	1137	1181	1090	1092	1192	1222
SAS	1363	1377	1403	1348	1329	1484	1496
EAP	1399	1427	1424	1395	1350	1520	1501
MENA	1676	1657	1630	1636	1571	1743	1702
LAC	1074	1085	1085	1055	1025	1107	1093
ECA	1042	1079	1106	1067	1067	1144	1165
NAM	812	812	808	799	771	863	850
Pulse supply (daily per-capita kilocalories)							
SSA	126	153	186	150	180	162	197
SAS	97	113	123	111	120	118	129
EAP	60	90	86	87	82	89	84
MENA	81	91	99	91	99	92	100
LAC	115	127	138	125	135	131	143
ECA	25	26	28	26	28	27	28
NAM	44	46	47	46	46	47	48
Fruit and vegetable supply (daily per-capita kilocalories)							
SSA	154	204	266	198	251	218	283
SAS	105	189	329	183	307	196	335
EAP	231	285	292	280	282	287	291
MENA	253	265	271	260	262	264	266
LAC	169	196	218	192	210	201	222
ECA	193	214	226	211	220	214	224
NAM	202	224	228	220	220	224	224
Root and tuber supply (daily per-capita kilocalories)							
SSA	408	426	434	415	415	461	469
SAS	54	69	74	64	66	72	75
EAP	147	154	148	149	141	159	153
MENA	78	80	82	75	73	82	82
LAC	115	110	105	107	101	113	107
ECA	162	158	155	152	147	159	155
NAM	104	102	101	97	94	103	100

1 Source: Authors. CC, climate change scenario; COMP, scenario of comprehensive investments in the agricultural sector for agricultural research and development, water management, and infrastructure; EAP, East Asia and Pacific; ECA, Europe and Central Asia; GDP, gross domestic product; LAC, Latin America and Caribbean; MENA, Middle East and North Africa; NAM, North America; NoCC, no climate change scenario; PPP, purchasing power parity; SAS, South Asia; SSA, Africa south of the Sahara.

In this study, estimates were made at the broad regional level based on available estimates of *H_xit_* (necessary for calibration of the regional error terms ε*_xi_*) from Gödecke et al. (14)Values are calculated and applied for East Asia and the Pacific (EAP), Europe and Central Asia (ECA), Latin America and the Caribbean (LAC), Middle East and North Africa (MENA), North America, South Asia (SAS), and Africa south of the Sahara (SSA).

Regional food system projections to 2050 were aggregated from country-level results from the IMPACT model, a quantitative foresight modeling framework for the global food system that generates scenario projections under different future conditions of the environment, socioeconomic factors, and other key drivers in the agricultural sector. The scenario outputs analyzed here are as published in Rosegrant et al. (17), which presented several climate scenarios along with an analysis of various scenarios of investment in the agricultural sector. Three scenarios from that study were selected to frame the current analysis, including reference scenarios with and without climate change—CC and NoCC, respectively—to provide a basis against which we can investigate the potential of a scenario with a comprehensive set of increased agricultural sector investments (COMP). Reference and adaptation scenario socioeconomic assumptions for population and GDP per capita follow the Shared Socioeconomic Pathway “2” (30), which is a middle-of-the-road projection that is standard for many global studies of the future of the agricultural sector. We also incorporate the potential for adjustments in GDP per capita by coupling the IMPACT model with a global general equilibrium model, GLOBE-Energy (31, 32), to simulate the full economy feedbacks on household income due to shocks and changes in the agricultural sector, which, in turn, affect food consumption levels (8, 33). The CC scenario follows a path of relatively strong climate change, known as Representative Concentration Pathway 8.5 (34) as modeled by the HadGEM global circulation model, a physical process model of the Earth's air, water, and land that simulates global change under changing greenhouse gas concentrations (35). Our CC scenario does not include the effects of carbon dioxide fertilization, which refers to enhanced plant growth resulting from increased levels of atmospheric carbon dioxide. Including carbon dioxide fertilization effects would partially offset the negative effects of climate change on crop productivity, although there remains uncertainty regarding the magnitude of this effect (36). This CC scenario was selected in conjunction with the NoCC scenario of a constant 2005 climate to represent upper and lower bounds for our modeling of pathways of the future climate.

In addition, the COMP scenario of comprehensive increased agricultural sector investment, which has climate change included as specified in the CC scenario, was incorporated to show the potential impact of policy actions that could be taken for adaptation to climate by improving sector productivity and resilience (and consequently DALYs) through *1*) increased agricultural research and development (R&D); *2*) water systems, water use efficiency (WUE), and management; and *3*) infrastructure improvement. Full details can be found in Rosegrant et al. (17), but here we provide a summary of the key scenario characteristics and basic outcomes.

### Reference scenarios (NoCC and CC)

The reference scenarios include trends of investments in agricultural R&D, irrigation expansion and water use management, and infrastructure that continue historical trajectories and incorporate expert judgment on nonlinear factors into a “business-as-usual” future for global food systems. Total current public spending in the agriculture sector in the developing world is US $86.4 billion per year and is projected to continue roughly at that level to midcentury (8, 17). The NoCC and CC share a common base of starting socioeconomic assumptions and baseline investments as described below with the distinction being that CC includes impacts of a relatively severe scenario of climate change (RCP 8.5 using the HadGEM), which, in general, sees global temperature increase, precipitation patterns shift, and agricultural commodity yields negatively affected (with regional- and country-level differentiation across all these dimensions).

### Baseline agricultural R&D

Agricultural productivity growth in the CC and NoCC scenarios is defined through commodity- and country-specific growth rates that incorporate historical trends and expert opinion on regional technological potential and crop yield gaps, developed with commodity experts from the CGIAR (a global research partnership of international agricultural research centers, formerly known as the Consultative Group for International Agricultural Research) and elsewhere. Meeting the productivity growth specified in the reference scenarios requires international agricultural R&D investments (in the CGIAR) estimated at an average of US $1.7 billion per year between 2015 and 2050 in constant 2005 dollars. In addition, average annual investment in national agricultural research systems (NARS) in developing countries is estimated to be US $6.4 billion per year under the reference scenario. The largest investments are projected in SSA, about 28% of the total average annual for the developing world. LAC, EAP, and MENA are each projected to require 19% to 22% of the total average annual for the developing world. In SSA, future required agricultural R&D investments in the reference scenarios are evenly split between NARS and the CGIAR, whereas NARS investments dominate in all other regions.

### Irrigation and water resource management

Trends in irrigation expansion and water management are also based on past trends, regional capacities, and expert opinion about future potentials. Total harvested area increases 18% from 2010 to 2050 with irrigated area growing faster than rainfed. For developing countries, investments for irrigation expansion are estimated at US $7.6 billion annually. Expansion is projected to be largest in EAP and SAS, but most investment expenditures will be focused in SSA because of greater need and higher costs in the region.

Water management is modeled here through WUE and improved soil-water capacity (ISW). WUE investments are critical to complement and accompany irrigation expansion around the world to reduce wastage and would total US $2.2 billion per year for the developing world, with most of these investments focused in EAP and SAS. ISW is also an important component of water management strategies on both irrigated and rainfed farmland and is estimated to cost US $1.3 billion annually for the developing world out to 2050.

### Infrastructure

Infrastructure expansion and maintenance in the reference scenarios, including rail, road, port, and electrification, are vital supports for the future food system in the developing world. Average annual investments are estimated at US $23.4 billion per year.

### Comprehensive investment scenario (COMP)

The COMP scenario includes increased investments—in addition to what is specified in the reference scenarios—in agricultural R&D, irrigation expansion and improved water resource management, and improved agricultural market efficiency achieved through increased infrastructure investments. In this modeling, the COMP scenario starts with CC and then adds in the components of increased investments noted below. The total incremental cost for COMP, on top of the estimated reference scenario cost, is US $25.5 billion annually from 2015 to 2050. The calculation of these costs is detailed in Rosegrant et al. (17) and Mason-D'Croz et al. (8). To understand this magnitude better, the total cost for COMP is 30% of current total public spending in the agriculture sector in the developing world (for the most recent year of data availability, between 2010 and 2014). A less than one-third increase in total public spending in agriculture is well within the margin of increases being proposed in current discussions about food system transformation between now and the Sustainable Development Goal target year of 2030 (37).

### Agricultural R&D for enhanced productivity

Agricultural R&D in the COMP scenario is focused on productivity improvements from increasing investments in the CGIAR, which works with components of the food system (crops, livestock, and fish) in major developing regions (SAS, SSA, LAC, EAP, and MENA). Yield increases are specified by commodity, region, and irrigated/rainfed production practices and can take the form of improved genetics, management/husbandry, or adoption of higher-yielding varieties, among many other options. We allow for a fluid interpretation of how yield gains can be achieved. Global costs for these investments are estimated at an average US $1.97 billion annually from 2015 to 2050 with the bulk, >84%, focused in SSA.

### Irrigation expansion and improved water resource management

Investments in irrigation expansion and WUE are combined into a unified investment in the COMP scenario. This is simulated by irrigated area expansion in developing countries to 2030 above levels in the reference scenarios that is designed to be neutral with respect to total land under agriculture. Irrigated area expands by 20 million hectares, which is offset by a 22-million-hectare reduction of rainfed agriculture. Increased yields on fewer irrigated hectares help keep total production equivalent to the more extensive rainfed area. Water use efficiency is modeled by assuming upgrades to existing irrigation systems to higher-efficiency technologies at the basin scale. Basin efficiencies are assumed to increase by 15 percentage points by 2030.

ISW simulates beneficial technologies that increase soil-water availability (e.g., no-till agriculture and water harvesting). Regional capacities for implementation of these technologies vary, with a maximum increase in effective precipitation of 5–15% by 2045.

Unit costs for these types of interventions include development, construction, and implementation. Irrigation expansion combined with WUE improvements in the COMP scenario is estimated to cost US $8.1 billion annually above the reference scenario. Expansion of irrigation will be most costly in SSA with WUE improvement costs that will be greatest in EAP. ISW across both irrigated and rainfed cropland carries an estimated cost of US $4.6 billion per year in the developing world.

### Improved infrastructure and market access

Infrastructure investments improve productivity and efficiency along the value chain to facilitate transport of commodities to intermediate producers and end consumers and lower marketing margins. This is achieved in the modeling framework by reducing costs imposed between farmgates and markets. Investments of US $10.8 billion per year across developing countries reduce price wedges between producer and consumer prices by 1 percentage point per year between 2015 and 2030.

### Yield, production, and area effects of COMP investment scenarios

Climate change generally slows yield growth that would be seen under the NoCC scenario. Across the developing world, climate change reduces average cereal yields by 6–9% by midcentury, with the strongest climate effects in SAS, LAC, and SSA. Although commodity production is projected to increase from 2010 to 2050 globally, this is slower with climate change compared with a NoCC future.

Under the COMP scenario, productivity improves substantially across the developing world, and the projection for total agricultural production expansion by 2030 and 2050 is 10% and 12%, respectively, compared with CC. SSA sees the largest increases at about double the average for the developing world. Yields improve most for cereals, roots and tubers, and pulses cultivated in developing countries. Cereal yields in SSA and MENA under COMP increase by ∼40% compared with CC in 2050, whereas LAC is projected to see a 35% increase. Area harvested, which combines both intensification and extensification of agricultural production, is projected to grow by 200 million hectares from 2010 to 2050.

This study did not involve any human subjects and did not require registration as a clinical trial or study with the NIH.

## Results

The relative burden of chronic and hidden hunger at the global level—and broken down into specific sources from the GBD—is presented in **Table 3**. Based on the values shown in that table, in 2010, the average person in the world experienced 0.0126 (12.60/1000) fewer healthy years of life due to chronic hunger and 0.01059 (10.59/1000) fewer healthy years due to hidden hunger. Attribution of these relative burdens to the component sources into future years was achieved by maintaining relative shares consistent with the data presented from the GBD in Gödecke et al. (14). This implementation for the IMPACT model can reasonably reproduce results reported from the GBD for 2010 (comparing historical and NoCC 2010 columns in Tables 3–*6*). Projections to 2030 and 2050 show the overall decline in the relative burden across the 3 IMPACT scenarios. Climate change, as shown by comparing CC with NoCC, slows this improvement and increases the relative burden by 1% to 2% in 2050 for chronic and hidden hunger. The optimistic, although feasible, COMP scenario of increased investment in the agricultural sector more than compensates for the impediments imposed by potential climate change by reducing these rates to below those found in the NoCC case.

**TABLE 3:** Disability-adjusted life years (DALYs) per 1000 capita due to chronic and hidden hunger at the global level^1^

			IMPACT			IMPACT		IMPACT		% change		% change	
	Historical		NoCC			CC		COMP		CC/NoCC		COMP/CC	
Characteristic	1990	2010	2010	2030	2050	2030	2050	2030	2050	2030	2050	2030	2050
Chronic hunger													
Global	*39.83*	*12.60*	*12.67*	*11.18*	*11.47*	*11.24*	*11.58*	*10.99*	*11.34*	*1*	*1*	*−2*	*−2*
Protein-energy malnutrition	2.30	1.40	1.32	1.17	1.20	1.17	1.21	1.15	1.18				
Childhood underweight	37.53	11.20	11.35	10.01	10.27	10.07	10.37	9.85	10.15				
Hidden hunger													
Global	*21.02*	*10.59*	*10.66*	*9.55*	*9.46*	*9.68*	*9.69*	*9.20*	*9.13*	*1*	*2*	*−5*	*−6*
Iodine deficiency	0.62	0.58	0.54	0.48	0.48	0.49	0.49	0.46	0.46				
Iron deficiency	9.82	6.97	6.81	6.10	6.04	6.18	6.19	5.87	5.83				
Vitamin A deficiency	5.88	1.67	1.88	1.68	1.67	1.70	1.71	1.62	1.61				
Zinc deficiency	4.62	1.32	1.40	1.25	1.24	1.27	1.27	1.20	1.20				
Other nutritional deficiencies	0.08	0.04	0.04	0.03	0.03	0.03	0.03	0.03	0.03				

1 Chronic and hidden hunger DALYs are due to protein-energy undernutrition and micronutrient deficiencies, respectively. Source: Authors and historical data from Gödecke et al. (14); published open access under the CC BY-NC-ND license (http://creativecommons.org/licenses/BY-NC-ND/4.0/). CC, climate change scenario; COMP, scenario of comprehensive investments in the agricultural sector for agricultural research and development, water management, and infrastructure; IMPACT, International Model for Policy Analysis of Agricultural Commodities and Trade; NoCC, no climate change scenario.

The total burden (in millions of DALYs) at the global level is presented in Table 4. Population growth is outstripping the declines in rates seen in Table 3. The total burden of DALYs due to chronic and hidden hunger is increasing globally to 2030 and 2050 by about 10 and 30 million, respectively, relative to 2010. As with the changes in incidence associated with climate change and agricultural sector investments in the COMP scenario, climate change worsens the total DALY burden, but increased investments can more than counteract this increase.

**TABLE 4:** Sum of disability-adjusted life years (DALYs) due to chronic and hidden hunger at the global level (millions)^1^

			IMPACT			IMPACT		IMPACT		% change		% change	
	Historical		NoCC			CC		COMP		CC/NoCC		COMP/CC	
Characteristic	1990	2010	2010	2030	2050	2030	2050	2030	2050	2030	2050	2030	2050
Chronic hunger													
Global	*209.7*	*86.9*	*87.2*	*92.6*	*105.3*	*93.1*	*106.3*	*91.0*	*104.1*	*1*	*1*	*−2*	*−2*
Protein-energy malnutrition	12.1	9.7	9.1	9.7	11.0	9.7	11.1	9.5	10.9				
Childhood underweight	197.5	77.3	78.1	82.9	94.3	83.4	95.3	81.5	93.3				
Hidden hunger													
Global	*110.6*	*73.1*	*73.4*	*79.1*	*86.9*	*80.1*	*89.0*	*76.1*	*83.9*	*1*	*2*	*−5*	*−6*
Iodine deficiency	3.3	4.0	3.7	4.0	4.4	4.0	4.5	3.8	4.2				
Iron deficiency	51.7	48.1	46.8	50.5	55.5	51.2	56.8	48.6	53.6				
Vitamin A deficiency	30.9	11.5	12.9	13.9	15.3	14.1	15.7	13.4	14.8				
Zinc deficiency	24.3	9.1	9.6	10.4	11.4	10.5	11.7	10.0	11.0				
Other nutritional deficiencies	0.4	0.3	0.3	0.3	0.3	0.3	0.3	0.3	0.3				

1 Chronic and hidden hunger DALYs are due to protein-energy undernutrition and micronutrient deficiencies, respectively. Source: Authors and historical data from Gödecke et al. (14); published open access under the CC BY-NC-ND license (http://creativecommons.org/licenses/BY-NC-ND/4.0/). CC, climate change scenario; COMP, scenario of comprehensive investments in the agricultural sector for agricultural research and development, water management, and infrastructure; IMPACT, International Model for Policy Analysis of Agricultural Commodities and Trade; NoCC, no climate change scenario.

Regional-level assessments for chronic and hidden hunger are presented in Tables 5 and 6. Again, although rates are declining—with climate change slowing progress and agricultural sector investments countervailing these setbacks—population growth quickly overcomes this progress.

**TABLE 5:** Disability-adjusted life years (DALYs) per 1,000 capita due to chronic and hidden hunger by region^1^

			IMPACT			IMPACT		IMPACT		% change:		% change:	
	Historical		NoCC			CC		COMP		CC/NoCC		COMP/CC	
Characteristic	1990	2010	2010	2030	2050	2030	2050	2030	2050	2030	2050	2030	2050
Chronic hunger													
Global	*39.83*	*12.60*	*12.67*	*11.18*	*11.47*	*11.24*	*11.58*	*10.99*	*11.34*	*1*	*1*	*−2*	*−2*
SSA	155.54	55.60	55.60	51.09	48.07	51.24	48.28	50.50	47.70	0	0	−1	−1
SAS	84.16	19.20	19.20	10.92	7.50	11.07	7.73	10.61	7.32	1	3	−4	−5
EAP	12.99	1.94	1.94	0.00	0.00	0.00	0.00	0.00	0.00	—	—	—	—
MENA	20.30	4.46	4.46	2.72	1.75	2.77	1.81	2.65	1.69	2	3	−4	−7
LAC	12.50	2.10	2.10	0.86	0.17	0.88	0.20	0.75	0.08	3	21	−15	−59
ECA	2.17	0.40	0.40	0.00	0.00	0.00	0.00	0.00	0.00	—	—	—	—
NAM	0.13	0.17	0.17	0.02	0.00	0.03	0.00	0.01	0.00	45	—	−66	—
Hidden hunger													
Global	*21.02*	*10.59*	*10.66*	*9.55*	*9.46*	*9.68*	*9.69*	*9.20*	*9.13*	*1*	*2*	*−5*	*−6*
SSA	67.64	29.82	29.82	27.56	25.50	27.77	25.86	26.92	24.95	1	1	−3	−4
SAS	39.59	16.94	16.94	13.77	12.37	13.93	12.65	13.28	11.93	1	2	−5	−6
EAP	9.51	4.19	4.19	1.51	0.92	1.63	1.14	1.28	0.74	8	22	−21	−34
MENA	14.41	8.78	8.78	7.31	6.29	7.39	6.45	6.99	5.97	1	2	−5	−7
LAC	15.12	6.56	6.56	5.28	4.40	5.36	4.53	5.04	4.17	2	3	−6	−8
ECA	4.84	3.53	3.53	2.84	2.31	2.90	2.41	2.68	2.15	2	4	−8	−11
NAM	0.43	0.33	0.33	0.16	0.13	0.20	0.20	0.03	0.00	24	49	−87	−100

1 Chronic and hidden hunger DALYs are due to protein-energy undernutrition and micronutrient deficiencies, respectively. Source: Authors and historical data from Gödecke et al. (14); published open access under the CC BY-NC-ND license (http://creativecommons.org/licenses/BY-NC-ND/4.0/). CC, climate change scenario; COMP, scenario of comprehensive investments in the agricultural sector for agricultural research and development, water management, and infrastructure; EAP, East Asia and Pacific; ECA, Europe and Central Asia; IMPACT, International Model for Policy Analysis of Agricultural Commodities and Trade; LAC, Latin America and Caribbean; MENA, Middle East and North Africa; NAM, North America; NoCC, no climate change scenario; SAS, South Asia; SSA, Africa south of the Sahara; —, denotes division by zero.

**TABLE 6:** Sum of disability-adjusted life years (DALYs) due to chronic and hidden hunger by region (millions)^1^

			IMPACT			IMPACT		IMPACT		% change:		% change:	
	Historical		NoCC			CC		COMP		CC/NoCC		COMP/CC	
Characteristic	1990	2010	2010	2030	2050	2030	2050	2030	2050	2030	2050	2030	2050
Chronic hunger													
Global	*209.7*	*86.9*	*87.2*	*92.6*	*105.3*	*93.1*	*106.3*	*91.0*	*104.1*	*1*	*1*	*−2*	*−2*
SSA	78.2	48.0	48.0	67.7	86.2	67.9	86.6	66.9	85.5	0	0	−1	−1
SAS	95.3	31.3	31.3	22.6	17.8	22.9	18.3	21.9	17.4	1	3	−4	−5
EAP	23.6	4.3	4.2	0.0	0.0	0.0	0.0	0.0	0.0	—	—	—	—
MENA	5.2	1.7	2.0	1.7	1.3	1.7	1.3	1.6	1.2	2	3	−4	−7
LAC	5.5	1.3	1.2	0.6	0.1	0.6	0.1	0.5	0.1	3	21	−15	−59
ECA	1.8	0.4	0.3	0.0	0.0	0.0	0.0	0.0	0.0	—	—	—	—
NAM	0.0	0.1	0.1	0.0	0.0	0.0	0.0	0.0	0.0	45	—	−66	—
Hidden hunger													
Global	*110.6*	*73.1*	*73.4*	*79.1*	*86.9*	*80.1*	*89.0*	*76.1*	*83.9*	*1*	*2*	*−5*	*−6*
SSA	34.0	25.8	25.7	36.5	45.7	36.8	46.4	35.7	44.7	1	1	−3	−4
SAS	44.8	27.6	27.6	28.5	29.3	28.8	30.0	27.5	28.3	1	2	−5	−6
EAP	17.2	9.2	9.2	3.5	2.1	3.8	2.5	3.0	1.7	8	22	−21	−34
MENA	3.7	3.4	4.0	4.4	4.5	4.5	4.6	4.2	4.3	1	2	−5	−7
LAC	6.7	3.9	3.8	3.6	3.3	3.7	3.4	3.5	3.1	2	3	−6	−8
ECA	4.1	3.1	2.9	2.4	2.0	2.5	2.1	2.3	1.8	2	4	−8	−11
NAM	0.1	0.1	0.1	0.1	0.1	0.1	0.1	0.0	0.0	24	49	−87	−100

1 Chronic and hidden hunger DALYs are due to protein-energy undernutrition and micronutrient deficiencies, respectively. Source: Authors and historical data from Gödecke et al. (14); published open access under the CC BY-NC-ND license (http://creativecommons.org/licenses/BY-NC-ND/4.0/). CC, climate change scenario; COMP, scenario of comprehensive investments in the agricultural sector for agricultural research and development, water management, and infrastructure; EAP, East Asia and Pacific; ECA, Europe and Central Asia; IMPACT, International Model for Policy Analysis of Agricultural Commodities and Trade; LAC, Latin America and Caribbean; MENA, Middle East and North Africa; NAM, North America; NoCC, no climate change scenario; SAS, South Asia; SSA, Africa south of the Sahara; —, denotes division by zero.

**FIGURE 1:**
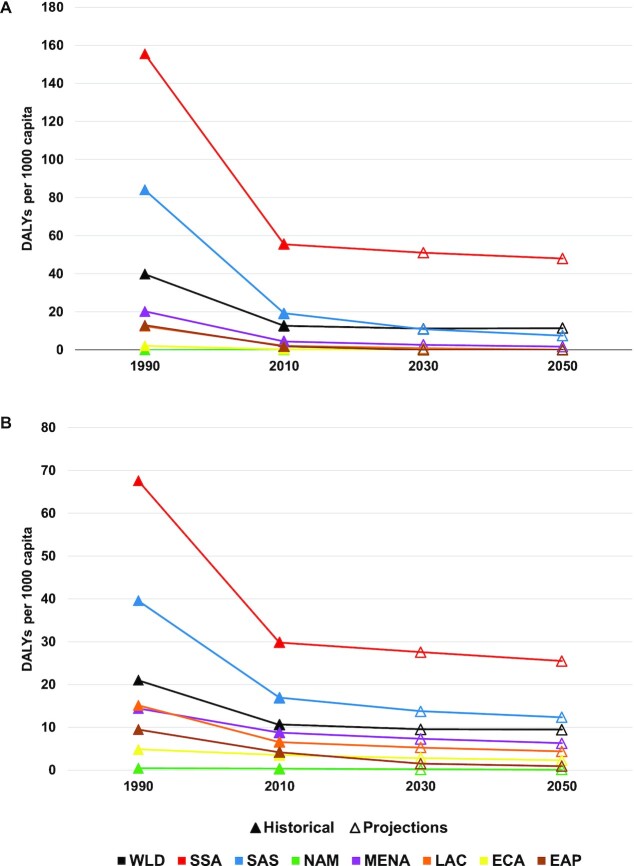
Regional and global incidence of disability-adjusted life years (DALYs) due to (A) chronic and (B) hidden hunger in the no climate change (NoCC) scenario. Chronic and hidden hunger DALYs are due to protein-energy undernutrition and micronutrient deficiencies, respectively. EAP, East Asia and Pacific; ECA, Europe and Central Asia; LAC, Latin America and Caribbean; MENA, Middle East and North Africa; NAM, North America; SAS, South Asia; SSA, Africa south of the Sahara; WLD, world. Source: Authors and historical data from Gödecke et al. (14); published open access under the CC BY-NC-ND license (http://creativecommons.org/licenses/BY-NC-ND/4.0/).

**FIGURE 2:**
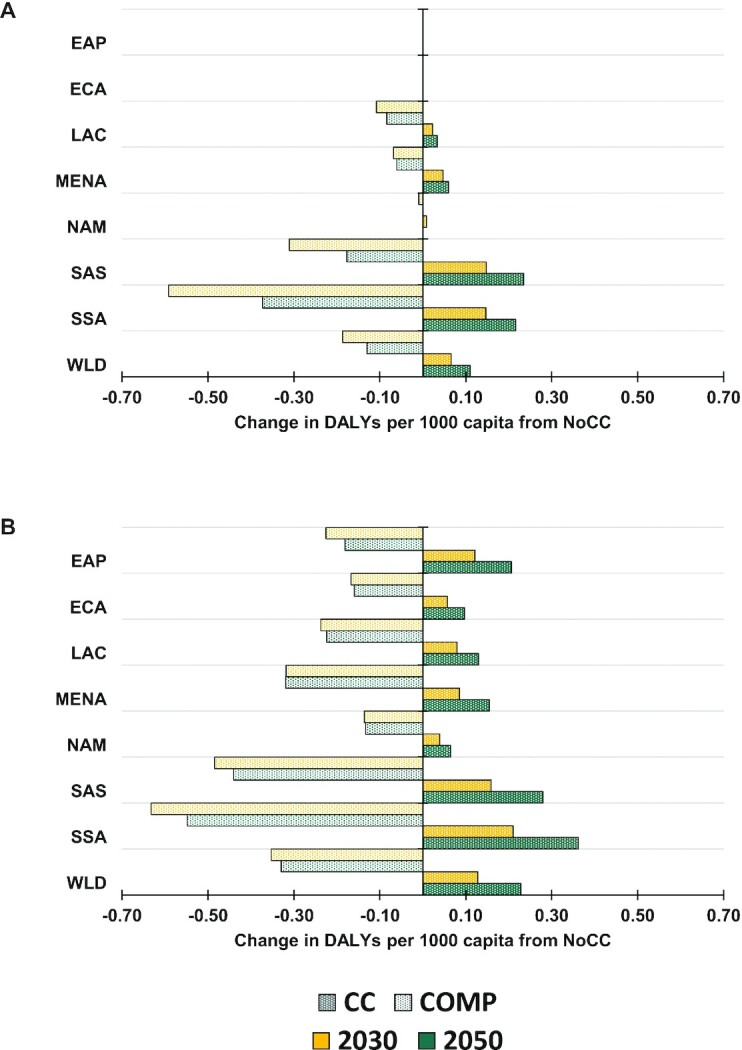
Regional and global change in (A) chronic and (B) hidden hunger incidence of disability-adjusted life years (DALYs) for climate change (CC) and investment scenarios (COMP) from the no climate change (NoCC) scenario. Chronic and hidden hunger DALYs are due to protein-energy undernutrition and micronutrient deficiencies, respectively. COMP, scenario of comprehensive investments in the agricultural sector for agricultural research and development, water management, and infrastructure; EAP, East Asia and Pacific; ECA, Europe and Central Asia; LAC, Latin America and Caribbean; MENA, Middle East and North Africa; NAM, North America; SAS, South Asia; SSA, Africa south of the Sahara; WLD, world. Source: Authors.

**FIGURE 3:**
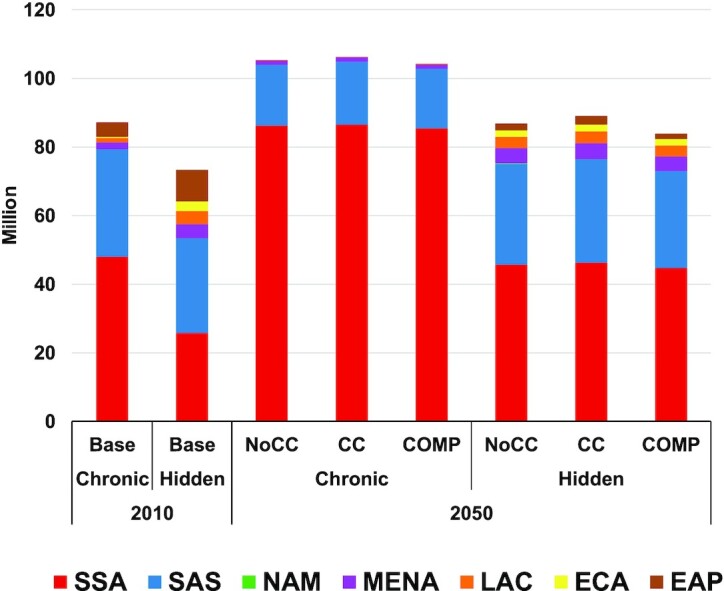
Regional breakdown of projections for total disability-adjusted life years due to chronic and hidden hunger by region in the base period (2010) and scenario projections for midcentury (2050) under no climate change (NoCC), climate change (CC), and increased agricultural sector investment (COMP). Chronic and hidden hunger DALYs are due to protein-energy undernutrition and micronutrient deficiencies, respectively. COMP, scenario of comprehensive investments in the agricultural sector for agricultural research and development, water management, and infrastructure; EAP, East Asia and Pacific; ECA, Europe and Central Asia; LAC, Latin America and Caribbean; MENA, Middle East and North Africa; NAM, North America; SAS, South Asia; SSA, Africa south of the Sahara. Source: Authors.

**Figure 1** shows the important progress in the reduction of the incidence of DALYs across regions in the NoCC scenario, with incidence rates cut by over half for both chronic and hidden hunger from 1990 to 2010. Further improvements, although at a much slower pace, continue out to midcentury. The changes in the per-capita DALY incidence due to chronic and hidden hunger for 2030 and 2050 under the CC and COMP scenarios from a NoCC future are shown in **Figure 2**. Climate change effects and the potential of offsetting these effects through agricultural sector investments are concentrated in SSA and SAS for chronic hunger. The CC and COMP effects on hidden hunger are spread out more globally but still greatest in SSA and SAS. **Figure 3** shows the regional outcomes of the dynamics between population growth and the burden of chronic and hidden hunger, with a challenging outlook for SSA alongside the important improvements in EAP. SSA is the primary source of increasing population that is driving the increase in the total DALY burden of both chronic and hidden hunger. SAS and MENA are also making minor contributions to the increase in the total DALY burden for hidden hunger. The situation for the total burden of chronic hunger in MENA is holding static, neither improving nor degrading.

**FIGURE 4:**
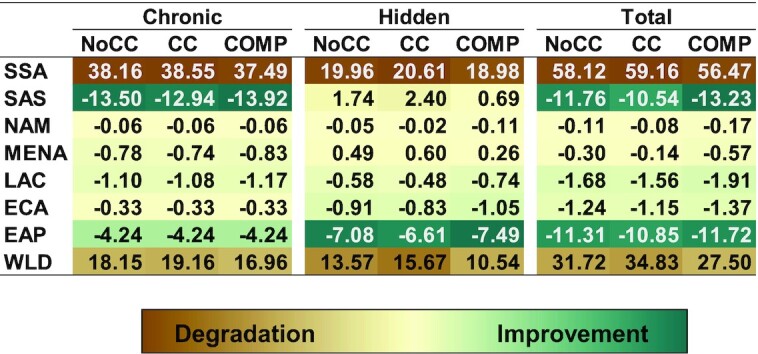
Heat map of change in millions of disability-adjusted life years due to chronic and hidden hunger from 2010 to 2050 under modeled scenarios from International Food Policy Research Institute's International Model for Policy Analysis of Agricultural Commodities and Trade model. Chronic and hidden hunger DALYs are due to protein-energy undernutrition and micronutrient deficiencies, respectively. CC, climate change scenario; COMP, scenario of comprehensive investments in the agricultural sector for agricultural research and development, water management, and infrastructure; EAP, East Asia and Pacific; ECA, Europe and Central Asia; LAC, Latin America and Caribbean; MENA, Middle East and North Africa; NAM, North America; NoCC, no climate change scenario; SAS, South Asia; SSA, Africa south of the Sahara; WLD, world. Source: Authors.

**Figure 4** shows the total change in DALYs due to chronic and hidden hunger from 2010 to 2050 in the IMPACT modeling scenarios. Chronic hunger is responsive to changes in SSA and SAS, whereas hidden hunger in SAS sees a stronger negative response (degradation). Climate change increases DALYs from chronic hunger by 1.0 million and hidden hunger by 2.1 million. The COMP scenario of increased investments in the agricultural sector more than offsets the impact of climate change and achieves levels of DALYs lower than a world without climate change (1.2 million less due to chronic hunger and 3.0 million less due to hidden hunger).

## Discussion and Conclusion

Previous work has demonstrated the association between a variety of diet and socioeconomic factors with past and present health outcomes as measured by DALYs. At the same time, many foresight modeling efforts have presented projections of the global food system with specifics regarding future levels of calorie and micronutrient availability and deficiencies under alternative future scenarios. Bringing these research fields together by incorporating econometric estimates of the relation of DALYs with projections of key food system indicators from a quantitative foresight modeling framework offers an important opportunity to advance the analysis of global food systems.

As used in this article, assessment of projections of DALYs due to both chronic and hidden hunger improves upon existing metrics focused primarily on calorie shortages by including the effects of key micronutrient deficiencies plus the longer-term and cumulative health outcomes of both chronic and hidden hunger. The DALY projections presented here are based on consumption across different key food types instead of just specific nutrients. Understanding how a broad health measure like DALYs could be affected by future food system evolution can help policymakers take action today to improve those outcomes. Using DALYs as a metric is appealing due to its universally comparable nature across a broad range of potential health issues. Rather than presenting policymakers with separate measures of the negative impacts of deficiencies in individual nutrients, these effects can be more readily compared or combined when all are reported in a common metric. In addition, the DALYs metric better captures health implications associated with different scenarios than the market-oriented outputs (e.g., prices and quantities) typically reported by economic models.

This work provides a means to generate these estimates for any number of future scenarios of change. This advance complements nutrient accounting (22) that sums nutrient availabilities across dietary components, comparison with healthy diet guidelines (38), and other ways to interpret the health implications of future diets (39, 40).

Calorie-focused measures—such as the prevalence of undernourishment (41), often referred to as “risk of hunger” (42, 8, 17), or simply a population count of people likely suffering from inadequacy of calorie availability from food systems model projections (43)—offer a simple perspective of potential shortcomings of the current and future food system. Although relatively established and robust, however, this approach can lead to a bias toward policy options that simply increase per-capita calorie availability to solve hunger issues. Previous analyses estimate that the climate change impact in 2050 on total developing world undernourished (compared with a NoCC scenario) varies from 6% to 45% increases (42), between a 15% decrease and a 50% increase (43), and a 13% to 20% increase (17). These earlier undernourishment metrics show generally stronger (and usually more negative) effects due to climate change than the present study estimates for DALYs. This is because DALYs are driven more strongly by changes in per-capita GDP than by changes in per-capita calorie availability, which is in line with findings from Hasegawa et al. (25).

We also tested specifications based on the other models in Gödecke et al. (14) that focused on projections of food availability (the more aggregated models 2 and 4 from Table 3) and found that the direction and magnitude of change in DALYs are largely similar across all 3 of these specifications (see **Supplemental Table 1**). Models 2 and 6 are especially close, with a difference of <1.5% in global total DALYs in 2050 across scenarios, with model 2 projecting slightly fewer DALYs due to hidden hunger than model 6 (0.2–2.3 million fewer globally, depending on the scenario in 2050). Using model 4 as the basis for the structural model projects fewer total global DALYs than both models 2 and 6, differing depending on region and scenario. Globally in 2050, model 4 projects 5–8% fewer total DALYs due to both chronic and hidden hunger compared with the model used here (model 6) and 8–11% fewer DALYs specifically due to chronic hunger.

Although the more aggregated models in Gödecke et al. (14) may have more attractive statistical significance for individual parameters, we feel it is important to build a single, consistent structural model that can use the key, commodity-specific projections coming from the quantitative foresight model. In this application, we have developed the structural model based on the econometric estimation of the importance of different diet drivers for the DALYs faced by regional populations around the world. Structural models enable development of policy experiments to help policymakers consider the impacts of different courses of action. These models are based on the best available data, but when statistically based parameters are not available, other expert-informed specifications may be used to explore alternative possible futures. It is important that structural models maintain relevance for policymakers, such as showing a more complete representation of a regional diet that enables development of counterfactual model-based policy experiments that are connected to the real-world situation faced by policymakers. Using model 2 or 4 as the basis for our analysis would mask important policy levers and emphasize simply increasing bulk per-capita calorie availability over balanced diets. Indeed, as shown in Table 1, we see that the most effective diet components to reduce chronic and hidden hunger are eggs, dairy, pulses, and roots and tubers, which could help steer policymakers away from a singular focus on increasing bulk energy availability through cereals alone, as has been the case in the past.

Some issues, however, could be addressed to improve this work. From the other models presented in Gödecke et al. (14), factors other than food supply generally have larger and more significant impacts on the number of DALYs due to the health burden of chronic and hidden hunger, such as other socioeconomic components, including health and sanitation, democratic governance and its stability, and education. These and other factors are currently accounted for in our modeling through the constant regional error terms.

Other food system components are also crucial. Increasing levels of atmospheric carbon dioxide are also affecting the nutrient content of foods, which further complicates hunger issues (21). Another important factor influencing food security and associated health impacts that is not adequately addressed when focusing on food supply is the role of food safety in reducing the sizable burden of foodborne disease (44). In addition, there are important links between food production and the spread of infectious diseases, particularly zoonotic diseases (45, 46). An integrated structural model—similar to Smith and Haddad (47)—could be estimated with an updated data set that would provide a more comprehensive set of results that would be an important advancement beyond the separate models presented in Gödecke et al. (14) and would provide more detailed inputs to improve modeling of future scenarios. The aggregated regions used in this analysis will only be useful for a certain set of decisionmakers, and country-level details are masked. Further work to develop country and subregional estimations of food system DALYs would be an important advancement. In addition, exploration of the extent of nonlinearities in the relation between DALYs and food supply would help bring more precise measurements to the connection between the food system and health outcomes.

Related to the further investigation of how the model could be specified, some parameter values (seen in Table 1) also seem counterintuitive, such as increased fish consumption driving an increase in both chronic and hidden hunger or fruits and vegetables being associated with increased hidden hunger. This could be resolved with a careful reanalysis of the latest data sets as just issued by Lenaerts and Demont (48) and perhaps a specification of alternative food groupings. There are several possible explanations: *1*) there is potential for multicollinearity in this model specification (where some variables could be highly correlated, such as many who eat a lot of fish also eat rice as their main staple), which could lead to some unexpected signs in the model parameters; *2*) there is also potential for aggregation issues from source data (e.g., SAS has lots of vegetable eaters but also high levels of hidden hunger, which may dominate the parameter value for fruits and vegetables); and *3*) it might also be the case where an aggregate fish category might be influenced by a nutritional anomaly we might not expect, as in Bogard et al. (49).

Overnutrition, the third component of the triple burden of malnutrition (50), would be an important dimension to add to the modeling, especially as this becomes a critical component of the dysfunction of food systems globally and in all regions.

All world regions have seen extraordinary progress in reducing the burden of undernutrition over the past several decades. Challenges for global and regional food systems remain, however, especially with respect to their overall resilience, as has been highlighted by our current crisis with COVID-19 (51). Akseer et al. (4) recently discussed implications of the COVID-19 pandemic on maternal and child health and nutrition, noting that new opportunities for mobilizing the agricultural sector to drive economic recovery are one approach to mitigate the adverse impact. The present work supports the contention that even in the face of climate change, we have the capacity to mitigate food insecurity and reduce disability associated with undernutrition through investment in agriculture. Within the agricultural sector, further improvements in the COMP scenario could be made by diversifying investments across different commodities and additional interventions. A broader array of investments beyond agriculture, such as in health systems directly, can accelerate further advancements for human health. Climate change is a rising stressor for food systems around the world, but as the scenarios explored here show, this is not an insurmountable challenge given sufficient political commitment (52) and investment in financing agricultural sector development. The connections among climate change, hunger, and other factors (21, 22) are complex and deserve further investigation beyond the brief presentation here.

An interdisciplinary approach that joins food systems with health outcomes and a comprehensive view of development can make the most of the strengths across disciplines to meet the nutritional needs of a growing population. Expanding populations can be a particular challenge, however, and other approaches to address undernutrition that go beyond food system approaches will be required. Complementary advancement in other societal dimensions important for DALYs, such as education, health, employment, and other dimensions, will help improve the situation in SSA and enable achievement of the “Zero Hunger” target of the Sustainable Development Goal 2. In the face of grand challenges for society to eliminate hunger and improve planetary and human health, thoughtful investments in the agricultural sector can make a difference, but there is a critical need to look beyond production of calories and toward more healthy diets and food systems. Modeling the impacts of alternative future pathways for DALYs can help inform the discussion.

## Supplementary Material

nqab101_Supplemental_File

## Data Availability

All data required for the analysis are included in the publication and/or available from cited sources within the article, and analysis was conducted in Microsoft Excel.

## Notes

Supported by USDA-ARS (NACA #58-8040-8-017) with additional support provided by the Wellcome Trust's Our Planet Our Health program (205212/Z/16/Z) to TBS, KDW, and SD through the Livestock, Environment and People project, but this work also builds on previous work supported by the CGIAR Research Program on Policies, Institutions, and Markets; the CGIAR Research Program on Climate Change, Agriculture and Food Security; USAID; and the Bill & Melinda Gates Foundation.

Supplemental Figure 1 and Supplemental Table 1 are available from the “Supplementary data” link in the online posting of the article and from the same link in the online table of contents at https://academic.oup.com/ajcn/.

Abbreviations used: CC, climate change reference scenario; COMP, comprehensive investment scenario; COVID-19, coronavirus disease 2019; DALY, disability-adjusted life year; EAP, East Asia and the Pacific; GBD, Global Burden of Disease; GDP, gross domestic product; IMPACT, International Model for Policy Analysis of Agricultural Commodities and Trade; ISW, improved soil-water capacity; LAC, Latin America and the Caribbean; MENA, Middle East and North Africa; NARS, national agricultural research systems; NoCC, no climate change reference scenario; R&D, research and development; SAS, South Asia; SSA, Africa south of the Sahara; WUE, water use efficiency.
